# Roles of Endoplasmic Reticulum Stress and Unfolded Protein Response Associated Genes in Seed Stratification and Bud Endodormancy during Chilling Accumulation in *Prunus persica*

**DOI:** 10.1371/journal.pone.0101808

**Published:** 2014-07-07

**Authors:** Xi Ling Fu, Wei Xiao, Dong Ling Wang, Min Chen, Qiu Ping Tan, Ling Li, Xiu De Chen, Dong Sheng Gao

**Affiliations:** 1 National Research Center for Apple Engineering and Technology, Shandong Agricultural University, Tai'an, Shandong, China; 2 State Key Laboratory of Crop Biology, Shandong Agricultural University, Tai'an, Shandong, China; 3 College of Horticulture Science and Engineering, Shandong Agricultural University, Tai'an, Shandong, China; University of Western Sydney, Australia

## Abstract

Dormancy mechanisms in seeds and buds arrest growth until environmental conditions are optimal for development. A genotype-specific period of chilling is usually required to release dormancy, but the underlying molecular mechanisms are still not fully understood. To discover transcriptional pathways associated with dormancy release common to seed stratification and bud endodormancy, we explored the chilling-dependent expression of 11 genes involved in endoplasmic reticulum stress and the unfolded protein response signal pathways. We propose that endoplasmic reticulum stress and the unfolded protein response impact on seed as well as bud germination and development by chilling-dependent mechanisms. The emerging discovery of similarities between seed stratification and bud endodormancy status indicate that these two processes are probably regulated by common endoplasmic reticulum stress and unfolded protein response signalling pathways. Clarification of regulatory pathways common to both seed and bud dormancy may enhance understanding of the mechanisms underlying dormancy and breeding programs may benefit from earlier prediction of chilling requirements for uniform blooming of novel genotypes of deciduous fruit tree species.

## Introduction

The peach (*Prunus persica*) originated in China and has been cultivated as an ornamental and economic fruit crop in Asia for about 3000 years because of its attractive flowers and delicious fruit. *Prunus persica* blooms earlier in spring compared with other deciduous fruit crops that usually require a long duration of chilling.

The dormancy and germination of seeds and buds are complex adaptive processes that are affected by a network of genes and environmental elements in tree species native to temperate and boreal regions [1]. Both types of dormancy are characterized by extremely low metabolic activities and a temporary insensitivity to growth-promoting signals. Dormancy is advantageous because it prevents the germination of intact viable seeds during temporarily advantageous conditions in an otherwise undesirable season [2]. Buds only attain ‘deep dormancy’ after a progressive process induces increasing intensity of dormancy during autumn [3]. Subsequently, a chilling accumulation requirement must be fulfilled for seed and bud dormancy release. Insufficient chilling can significantly impact on the time of seed germination and bud break, the duration of blooming, and fruit quality [4]. This phenomenon may result in major economic losses to the agricultural industry.

To understand the mechanisms underlying dormancy, much progress has been made in identification of the genes associated with dormancy. In this regard, a variety of woody species, including peach [5], Japanese apricot [6], grape [7], poplar [8], and raspberry [9], have been studied. Nevertheless, the mechanisms responsible for dormancy in deciduous fruit trees remain to be elucidated.

Proteomic analysis has become an extremely valuable approach with which to investigate the molecular pathways and identify the proteins involved in seed dormancy [10], [11]. Proteins that are secreted to the cell surface first enter the endoplasmic reticulum (ER), where they are folded and assembled before their delivery to other compartments in the endomembrane system. A conserved signalling pathway termed the unfolded protein response (UPR) monitors the protein-folding capacity in the ER lumen and transduces information on perturbations in the protein-folding status to the nucleus and cytosol [12]. Plants maintain a balance between protein-folding and -degradation capacity in line with demand either by enhancing the protein-folding or -degradation machinery, or by slowing protein production [13].

Most previous studies of UPR have focused on yeast and mammalian cells. The UPR signalling pathways in mammalian cells comprise three branches that involve different ER stress transducers and sense the accumulation of misfolded or unfolded proteins and target the stress response genes. One pathway is regulated by the mammalian homolog of inositol-requiring enzyme-1 (IRE1), which splices the mRNA of bZIP-like transcription factor X-box binding protein 1 (XBP1). A second pathway is mediated by activating transcription factor 6 (ATF6), which is transported to the Golgi to be processed by site 1 and site 2 proteases (S1P and S2P). In the third pathway translation is regulated by RNA-activated protein kinase (PKR) - like ER kinase (PERK) [14].

The UPR in plants involves the transcriptional regulation of genes that function in protein folding and degradation [15]. In plants only two arms of the UPR signalling pathway have been identified: one arm involves the proteolytic processing of membrane-associated basic leucine zipper domain (bZIP) transcription factors (ATFs) and the second arm involves the RNA splicing factor IRE1 [16]. PERK has not been detected in *Arabidopsis* [16], [17]. In *Arabidopsis*, ER stress is sensed and stress signals are transduced by the membrane-bound IRE1-like (IRE1a and IRE1b) and ATF6-like (bZIP28) transducers. Plant cells contain IRE1a and IRE1b. IRE1a and IRE1b are similar in structure and function to mammalian counterparts, which target bZIP60 mRNA [18], [19]. In response to ER stress, protein kinase and endoribonuclease activities of IRE1a are activated through the dissociation of binding immunoglobulin protein (BiP) from IRE1a and subsequent dimerization. Activated IRE1a removes an unconventional intron from unspliced bZIP60 mRNA in the cytosol. The bZIP60 translocates to the nucleus and controls transcription of the genes that encode ER chaperones. In response to ER stress, IRE1b induces translational repression through 28S ribosomal RNA cleavage. Similar to ATF6 in response to ER stress, the bZIP28 transcription factor is activated and relocates from the ER to Golgi where it is processed by S1P and S2P successively [20]–[22]. The bZIP28 assembles a larger transcriptional activation complex by interaction with the heterotrimeric CCAAT binding factors composed of subunits NF-YA4, NF-YB3, and NF-YC2. Thus, IRE1-bZIP60 and bZIP28-NF-Y UPR signalling pathways are known in plants.

Recent studies show that in plants the UPR is closely associated with response to adverse environmental stresses, such as salt stress [20], [23], heat stress [24]–[26], and drought stress [27], [28]. Thus, ER stress and UPR genes are involved in the response to abiotic stress. However, the genotype-specific period of seed stratification and bud endodormancy are elicited by ER stress and the UPR by molecular mechanisms that are poorly understood.

To elucidate characteristics common to seed and bud dormancy, we explored the expression of ER stress- and UPR-associated genes involved in transcriptional regulation during chilling of seeds and buds in peach. Clarification of the regulatory pathways of ER stress and the UPR in both seeds and buds will enhance understanding of the mechanisms of dormancy and may benefit breeding programs through earlier prediction of chilling requirements for uniform blooming of novel genotypes of deciduous fruit tree species.

## Materials and Methods

### Plant material

Trees of peach (*Prunus persica* (L.) Batsch var. *nectarina* (Suckow) C. K. Schneid.) were cultivated for five years under standard agricultural practices at the horticulture experimental station of Shandong Agricultural University, Tai'an, China. Experiments were carried out between June 2011 and February 2012.

### Seed sampling

Two hundred and forty ripe fruits were collected from 60 distinct trees (4 fruits each tree, respectively) on 26 June, 2011, which were divided randomly into three groups. The endocarp was dissected from the fruit immediately. Seeds were sterilized and flamed with alcohol, and then the coats were removed under sterile conditions. Embryos were cultured on 20 ml aliquots of sterile WPM medium solidified with 0.8% bacteriological agar in culture tubes. The tubes were stored at 4°C under continuous darkness for 0, 15, 30, 45, and 60 days, respectively [29]. Thirty randomly selected, untreated embryos were frozen in liquid nitrogen and stored at −80°C for RNA extraction and quantitative real time polymerase chain reaction (qRT-PCR) analysis.

### Bud sampling

Buds were sampled before leaf abscission, and during the dormancy and dormancy-release periods, on 15 and 23 October, 1, 8, 15 and 23 November, 1, 8, 15 and 23 December, and 1 January. At each time point, 60 flower buds with the scales removed were harvested from first-year branches of different vigorous individual trees, immediately frozen in liquid nitrogen, and stored at −80°C for RNA extraction and qRT - PCR analysis.

### Measurement of bud endodormancy status

Bud endodormancy was measured on 120 first-year branches incubated in 5% sucrose solution after leaf abscission on 15 and 23 October, 1 and 15 November, 1, 15 and 23 December, and 1 January. Trials were conducted in a completely randomized design with three replicates of 40 cuttings per treatment. The branches were incubated in a growth chamber under a daily cycle of 25°C with artificial fluorescent light (200 mol µm^−2^ s^−1^) for 16 h and 18°C in darkness for 8 h with constant 70% relative humidity. Bud break was considered to have occurred when a green shoot tip was observed among the bud scales. The mean time to bud break (MTB) was calculated as described by Leida *et al*. [30]. The period before the date that corresponded to an MTB interval of 6 weeks was considered to represent paradormancy, and the same interval after this date represented endodormancy. The basal ends of the shoots were cut weekly, and sucrose solution was replaced daily. The number of sprouting buds was recorded weekly. The time to bud break of a group of shoots was the time in days required for at least 50% of the flower buds to break. The results were expressed as the mean time to sprout for the three replicates.

### Genes associated with ER stress and UPR in stratified seeds and buds

To explore changes in gene expression during seed stratification and bud endodomancy under chilling treatment, the expression of ER stress-related genes in experimentally chilled embryos and buds were analysed by qRT-PCR. BiP, a member of the HSP70 family, is the most abundant chaperone in the ER and includes BiP1 and BiP2. Ppa002489m and ppa002572m code for putative BiP1 and BiP2 proteins in peach, respectively. The relative expression of transcripts that code for IRE1a-like (peach transcript model ppa001128m), IRE1b-like (ppa001418m), bZIP28-like (ppa002181m), bZIP60-like (ppa008311m), S1P-like (ppa000662m), and S2P-like (ppa024254m) protein in stratified seeds and buds during endodormancy were analysed. The bZIP28 interacts with the heterotrimeric CCAAT binding factors composed of subunits NF-YA4, NF-YB3, and NF-YC2, therefore expression of *NF*-*YA4*-like (ppa011627m), *NF*-*YB3*-like (ppa011714m) and *NF*-*YC2*-like (ppa010147m) transcripts were also analysed.

### Isolation of RNA and qRT-PCR analysis

Total RNA from 100 mg seeds (at least four seeds every time) with their coats removed or 300 mg buds was isolated using the RNeasy Plus Mini Kit (Qiagen, Valencia, CA, USA) in accordance with the manufacturer's instructions. Chloroform (200 µl) was included in the extraction buffer for seeds. Three duplicates of approximately 9 µg total RNA extracts was reverse-transcribed with the SuperScript III First-Strand Synthesis System for RT-PCR (Invitrogen, Carlsbad, CA, USA) in a total volume of 20 µl. The qRT-PCR was performed with SYBR Premix Ex Taq (TaKaRa Biotechnology, Dalian, China). Composition of the reaction solution and conditions followed the manufacturer's instructions. The primers employed are listed in Table 1. The cycling protocol comprised 10 min at 95°C, then 40 cycles of 15 s at 95°C for denaturation and 1 min at 60°C for annealing and extension. Specificity of the PCR was assessed by the presence of a single peak in the dissociation curve after the amplification and by size estimation of the amplified product. The comparative threshold cycle C_T_ (2^−ΔΔCT^) method was used to quantify cDNAs with amplification efficiencies equivalent to that of the reference actin gene. The standard curve regression was applied when amplification efficiencies were not equivalent to that of the reference actin gene. Results presented are the average of three independent biological replicates repeated three times.

**Table 1:** Sequences of primer pairs used for qRT-PCR.

Accession number (Gene)	Forward Primer (5′ - 3′)	Reverse Primer (5′ - 3′)
ppa002489m (*BiP1*-like)	ACCAGGGTAACCGTATCAC	GTCCCTCTGGACCTCTTT
ppa002572m (*BiP2*-like)	TTGCTGGGCTCAATGTGG	ATAGCTGCCGCTGTAGGC
ppa001128m (*IRE1a*-like)	GTAGTTGGAGAGGAAGAT	ATAATAGAGGTGACAGGAA
ppa001418m (*IRE1b*-like)	ATATTGTCCGTTGGTATGG	GTTCCTGATTCTTGGTGAT
ppa002181m (*bZIP28*-like)	AGTGAGTGACGAAGGCAACG	CTTATCCTCAAGCTCCTCGACATAA
ppa008311m (*bZIP60*-like)	AGGATGCAGCGGTGAGAT	GCAGCACTGGAGCAAACG
ppa000662m (*S1P*-like)	TTGTTATTGGCTCTTGAGGAA	ACGGTAGTTGGTTGTCTTC
ppa024254m (*S2P*-like)	GCCTAACTCTATGATGGA	TTCTGCTTGGATGATTATG
ppa011627m (*NF-YA4*-like)	GTTCTGATAATAGTGAATCTGATG	GTCCTGTTCCAACTTGTG
ppa011714m (*NF-YB3*-like)	GCATCAGCATCAGCATCA	AACCCAACTCCACTCACA
ppa010147m (*NF-YC2*-like)	CAACAACTTCAGCAACAG	TCCTCATCAGCCTTCATA
ppa002399m (*ACTIN*)	GTTATTCTTCATCGGCGTCTTCG	CTTCACCATTCCAGTTCCATTGTC

### Statistical analysis

Statistical analysis was performed using SPSS for Windows version 19 (SPSS, Chicago, IL, USA). Categorical variables were expressed as frequencies, and percentages and continuous variables as the mean ± standard deviation, as appropriate. The data were analysed by ANOVA and, when appropriate, Duncan's test was used. A significance level of p<0.05 was applied.

## Results

### Measurement of bud endodormancy status

**Figure 1:**
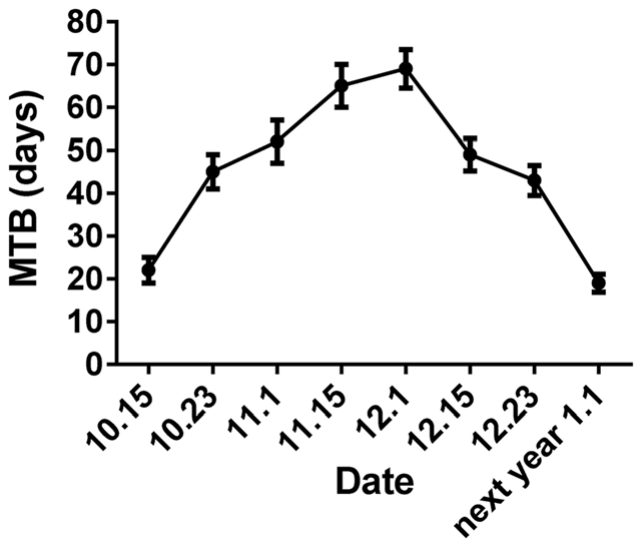
Progression of bud dormancy. The data indicate variation in mean time to bud break (MTB) values during dormancy from 15 October 2011 to 1 January 2012. Values are means (*n* = 3) and error bars represent the standard deviation.

Seasonal progression of bud MTB followed an oscillating pattern during the experimental period (Figure 1). A marked increase in MTB occurred in early winter until late November, when the highest MTB value was recorded. A slight decrease in MTB was observed in early December and thereafter MTB continued to decrease in December and January until dormancy release. The minimum MTB observed was acquired by early January, just before blooming. The interval of 6 weeks in MTB before 23 October was considered to represent paradormancy, and the same interval in MTB between 23 October and 23 November represented endodormancy.

### BiP transcript accumulation and IRE1-bZIP60 pathway activation

**Figure 2:**
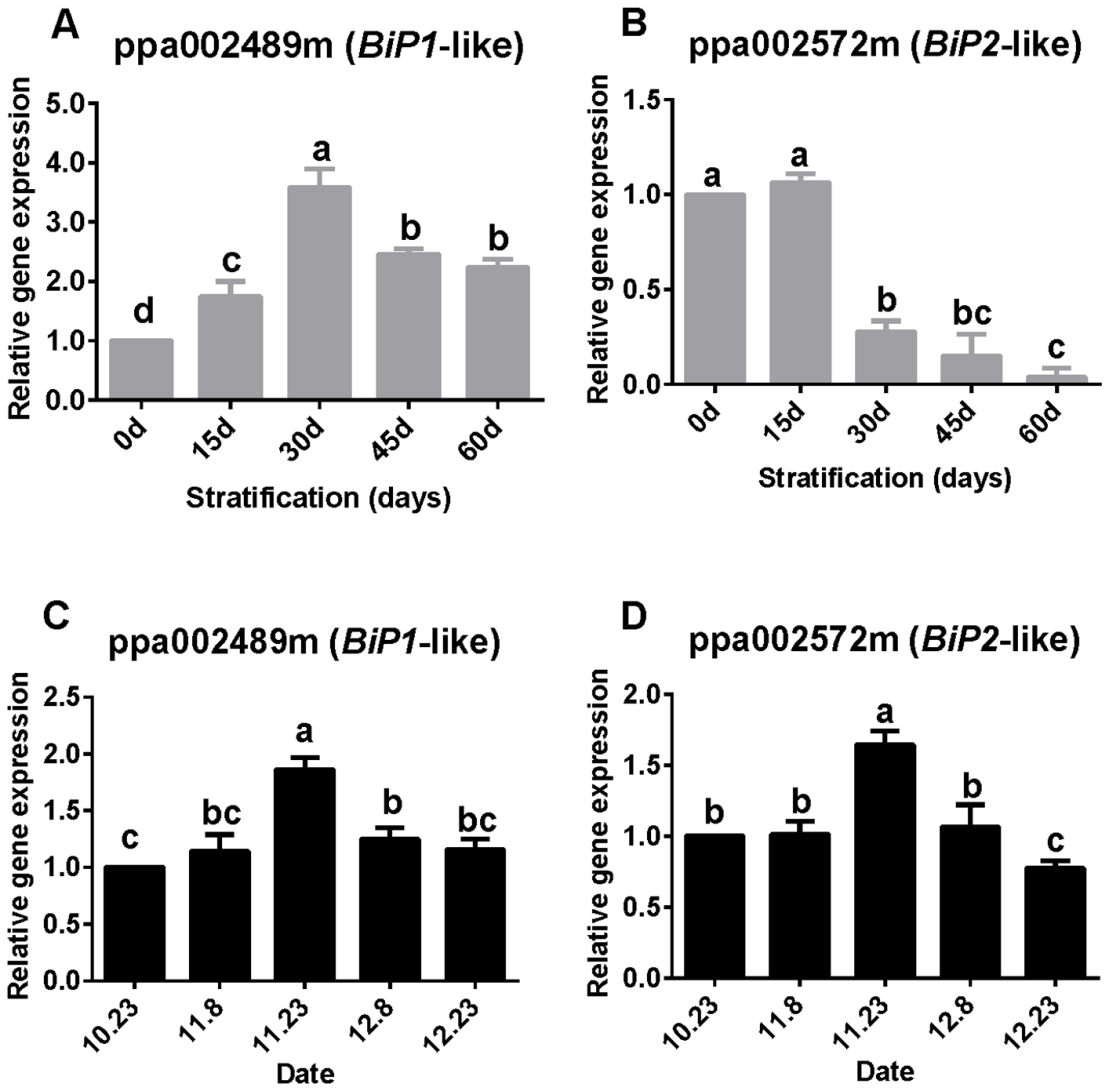
Relative expression levels of *BiP1*-like and *BiP2*-like. Expression was measured in seeds (A, B) and buds (C, D). Expression levels were normalized against that of the actin gene. Values are means (*n* = 9) and error bars represent the standard deviation. Different letters above bars indicate a significant difference among chilling periods according to ANOVA and Duncan's test.

During seed dormancy, *BiP1*-like transcripts accumulated during the first month and attained a peak that was three-fold higher than the baseline level (Figure 2A). Thereafter, the expression level was lower but remained relatively stable until dormancy release. In contrast, expression of *BiP2*-like transcripts drastically declined after 15 days and subsequently remained stable at an extremely low level. Expression of *BiP1*-like and *BiP2*-like transcripts in dormant buds showed similar trends to those observed in stratified seeds, with transcript expression showing a significant increase during the first month and subsequently decreasing to a lower, significantly different level (Figure 2C, D).

**Figure 3:**
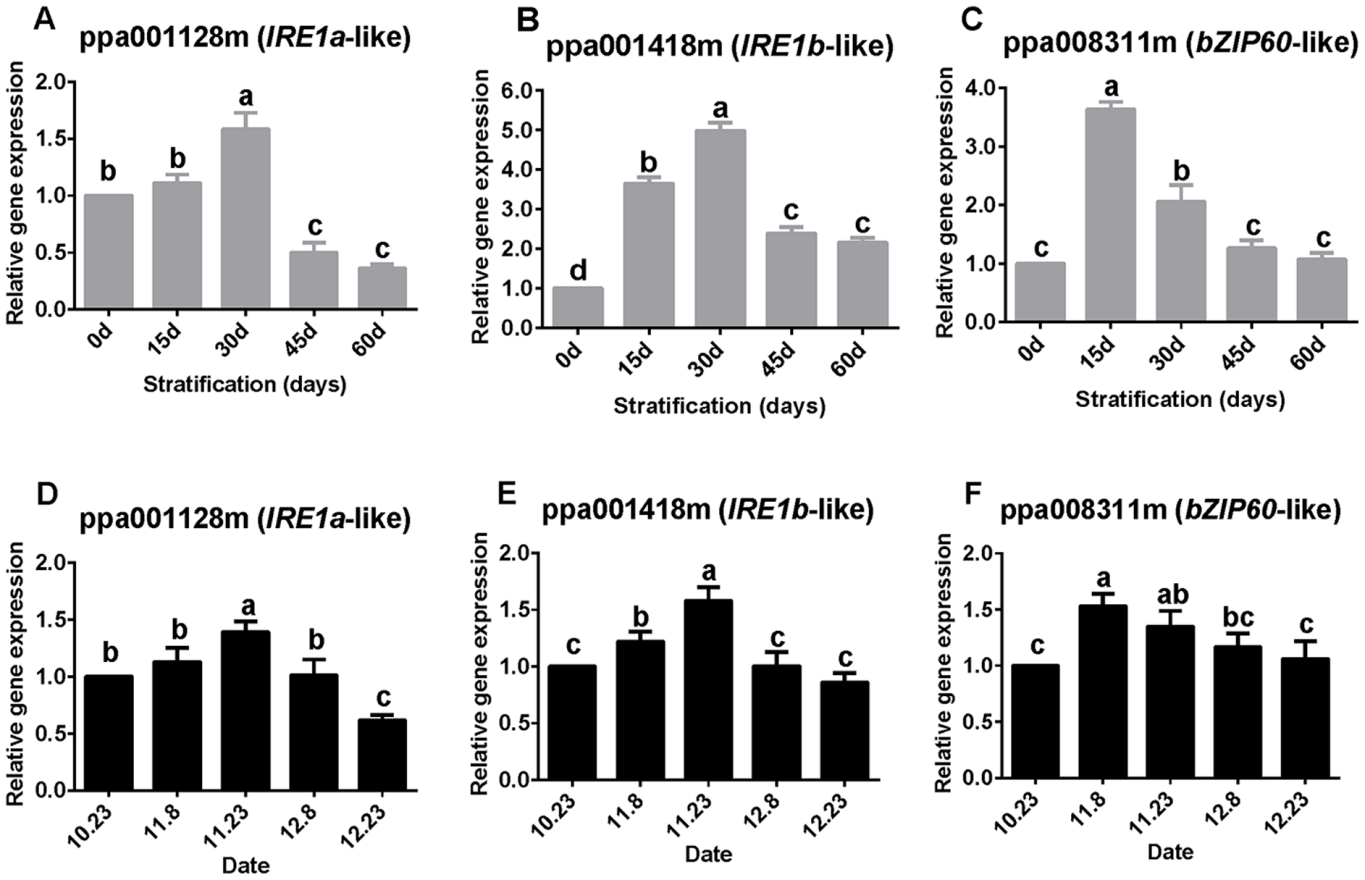
Relative expression levels *IRE1a*-like, *IRE1b*-like, and *bZIP60*-like. Expression was measured in seeds (A–C) and buds (D–F). Expression levels were normalized against that of the actin gene. Values are means (*n* = 9) and error bars represent the standard deviation. Different letters above bars indicate a significant difference among chilling periods according to ANOVA and Duncan's test.

Expression of *IRE1a*-like and *IRE1b*-like transcripts significantly increased within the first 30 days of chilling (Figure 3A, B). Expression of both genes subsequently decreased significantly. Transcription of both genes during bud dormancy showed the same trends as those observed during seed dormancy (Figure 3D, E). A significant decrease in expression of *IRE1a*-like and *IRE1b*-like in buds was observed after 23 November.

In seeds, *bZIP60*-like transcription was significantly up-regulated and maximum expression was observed after chilling for 15 days. The peak expression level was 3.63-fold higher than the baseline level (Figure 3C). Subsequently, the expression level declined and after chilling treatment for 45 days did not differ significantly from the baseline level. Expression of in buds showed similar trends, although the changes were less marked compared with those observed in seeds (Figure 3F).

### Up-regulation of bZIP28-NF-Y transcription factors

**Figure 4:**
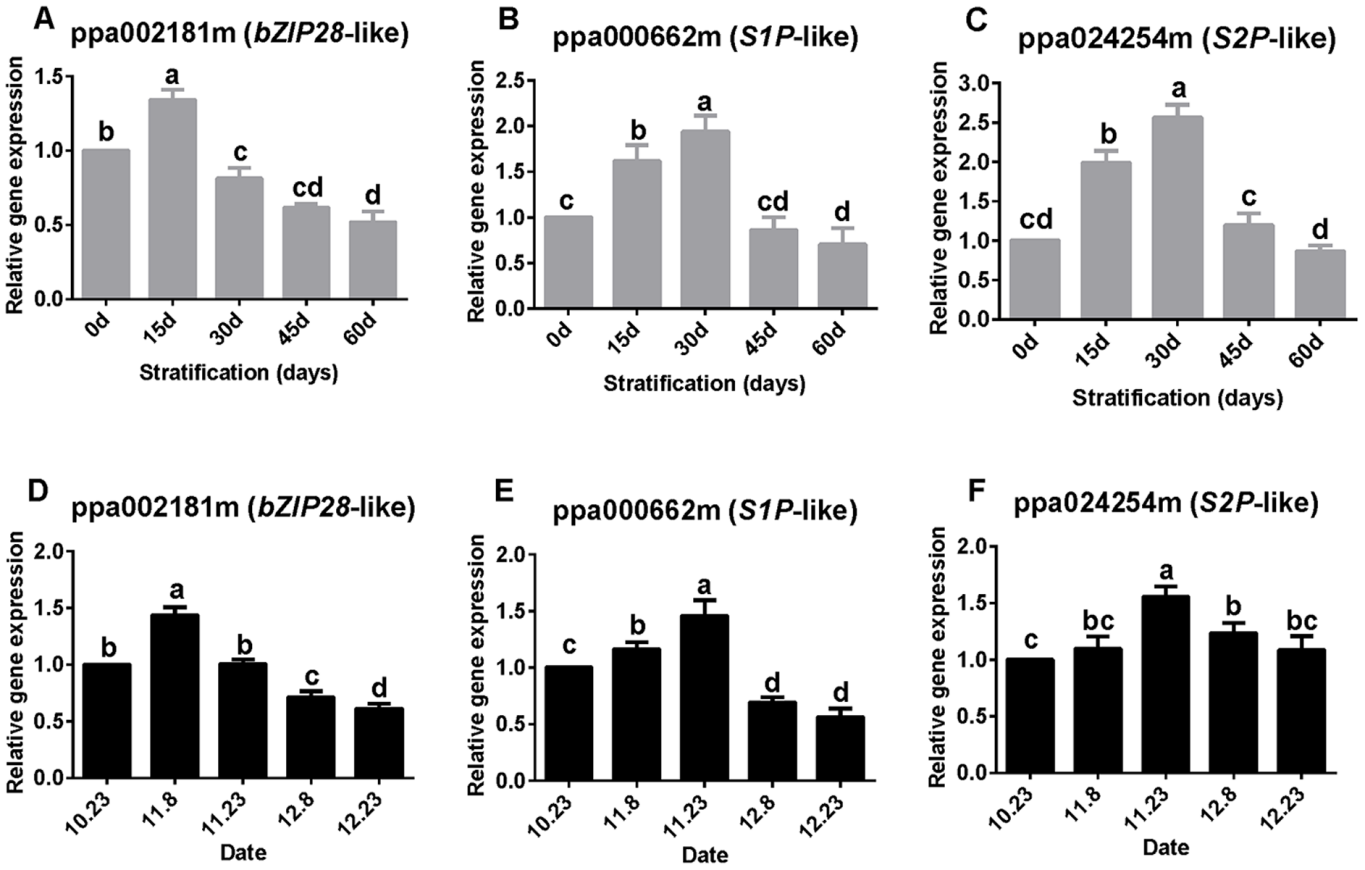
Relative expression levels of *bZIP28*-like, *S1P*-like, and *S2P*-like. Expression was measured in seeds (A–C) and buds (D–F). Expression levels were normalized against that of the actin gene. Values are means (*n* = 9) and error bars represent the standard deviation. Different letters above bars indicate a significant difference among chilling periods according to ANOVA and Duncan's test.

Expression of *bZIP28*-like in seeds and buds was significantly up-regulated after 15 days of chilling, but thereafter *bZIP28*-like was down-regulated and the expression level was significantly lower than the baseline level (Figure 4A, D).

A gradual increase in *S1P*-like and *S2P*-like expression levels was observed after deep dormancy was formed during the first 30 days of chilling in both seeds and buds. The peak expression level was significantly higher than the baseline level (0 day, 23 October) (Figure 4B, C, E, F). Subsequently, expression decreased to a level similar to, or significantly lower than, the baseline level.

**Figure 5:**
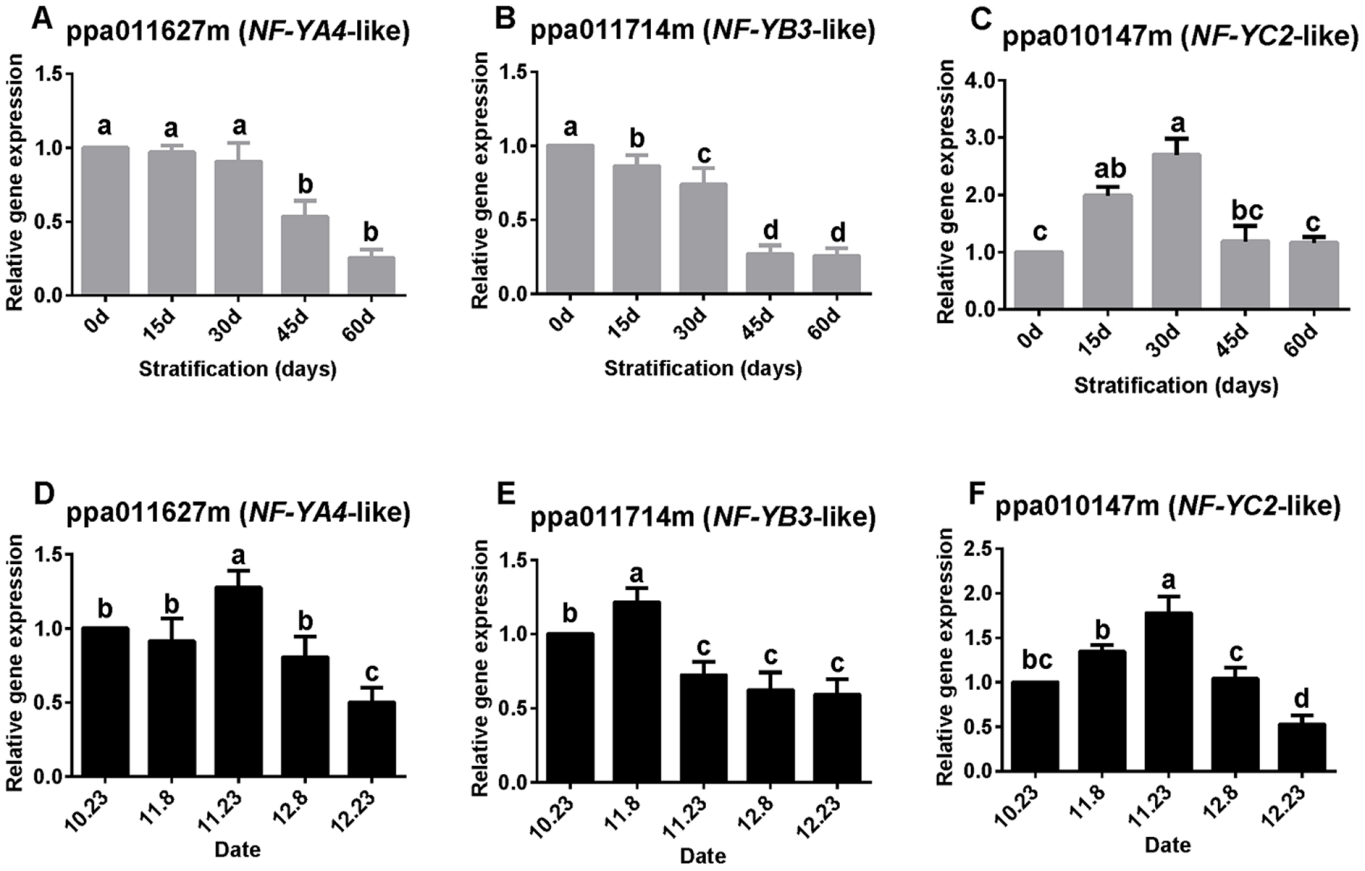
Relative expression levels of *NF-YA4*-like, *NF-YB3*-like, and *NF-YC2*-like. Expression was measured in seeds (A–C) and buds (D–F). Expression levels were normalized against that of the actin gene. Values are means (*n* = 9) and error bars represent the standard deviation. Different letters above bars indicate a significant difference among chilling periods according to ANOVA and Duncan's test.

In seeds *NF*-*YA4*-like and *NF*-*YB3*-like transcripts showed a continuous, significant decline in expression from the baseline level throughout the experimental period. After chilling for 60 days, the relative expression level was only about one-fourth that of the baseline (Figure 5A, B). In contrast, the *NF*-*YC2*-like expression level in seeds increased significantly to a maximum after chilling for 30 days, and thereafter declined to a level similar to the initial baseline values (Figure 5C).

The changes in *NF*-*YC2*-like expression in buds were similar to those observed in seeds (Figure 5F). However, trends in the expression of *NF*-*YA4*-like and *NF*-*YB3*-like transcripts in buds differed from those observed in seeds. *NF*-*YA4*-like and *NF*-*YB3*-like transcripts were both up-regulated but at different time points (*NF*-*YA4*-like, 23 November; *NF*-*YB3*-like, 8 November) (Figure 5D, E).

## Discussion

Dormancy of seeds and buds in deciduous fruit trees are complex phenomena that provide a protective mechanism against the impact of low winter temperatures on plant survival, development and architecture [31]. The ability to manipulate these processes is important to increase the yield and seasonal availability of many fruit crops. In many cases, release of dormancy results in increased cell division and changes in developmental programs. Much can be learned about dormancy regulation by identifying interactions of signals in these crucial processes.

Studies of the regulation of bud dormancy have resulted in some of the most fundamental discoveries in plant science. Related studies of plant growth and shoot development have identified many genes involved in meristem initiation and organ formation, and many genes and signals that control cell division in plants [4]. Despite these findings, gaps remain in our knowledge of the induction and release of bud and seed dormancy. Endogenous dormancy, which is regulated by endogenous physiological factors, plays a pivotal role in bud and seed dormancy. The temperature in autumn and early winter contributes to the intensity and progression of dormancy. Analogous observations are reported in apple, in which the chilling accumulation affects the progression of bud dormancy [32]. Chilling accumulation has long been considered to be the major factor leading to endogenous dormancy [33]. The present research is aimed at determining whether the fundamental process of dormancy release in seeds and buds dependent on chilling accumulation is regulated by a common signalling pathway.

Endoplasmic reticulum stress agents that affect Ca^2+^ homeostatic balance are surrogates for environmental stresses, and all of these agents induce the UPR. As yet, there is no clear understanding as to how endogenous dormancy induces ER stress and the UPR in plants and how the membrane-associated transcription factor system discriminates during dormancy. BiP has been demonstrated to act as a multifunctional protein in animal systems [34]. In addition to its role as molecular chaperone, in mammalian cells BiP has been shown to function as a sensor of alterations in the ER environment that activate the cytoprotective UPR [34]. Like BiP from animal systems, plant BiP has been shown to regulate UPR negatively [35], which have been identified in a variety of processes, such as the regulation of endosperm proliferation in *Arabidopsis thaliana* and the accumulation of seed storage proteins in rice, and are up-regulated in response to abiotic stresses such as salt, drought and other ER stress agents [17], [19], [21], [36]–[39]. Therefore, it will provide an improved understanding of folding capacity in the ER and more accurate assessment of the stress status of the plant by monitoring *BiP* transcription [27], [37], [40]. In the present study, two marker genes of ER stress, *BiP1* and *BiP2*, were up-regulated by fulfilment of the chilling requirement. BiP accomplishes two main functions. During accumulation of unfolded or misfolded proteins induced by endodormancy in the ER, BiP appears to alleviate the stress by binding to misfolded proteins in the ER to prevent their abnormal aggregation during refolding processes. Secondly, BiP is one of the main transcription targets of active bZIP transcription factors [20], [21], [41]. BiP regulates the activity of the ER stress sensors or transducers, bZIP28 and bZIP60, as well as IRE1a and IRE1b, through targeting increased expression of the chaperones of BiP or decreasing the transcriptional expression of unfolded or misfolded proteins. Collectively, the UPR is a critical approach to establish homeostasis and also plays a crucial role in mediating the release of endodormancy by processing misfolded or unfolded proteins.

The present qRT-PCR analysis revealed a parallel pattern of gene expression related to dormancy in seeds and buds. Most of the genes down-regulated during bud endodormancy release after fulfilment of the chilling requirement were also repressed by cold stratification in seeds. This suggests that common regulatory signalling pathways are involved in the mechanisms of endogenous dormancy and its release in seeds and buds.

The transcriptional similarities between bud and seed dormancy highlighted in the present study may also be relevant for plant breeding purposes. The selection of early- and late-flowering genotypes from a segregating population usually requires the arduous evaluation of large collections of individuals, which could be improved by previous selection of the desirable trait at the seed level. Previous studies found a positive correlation between the chilling requirements for seed germination and blooming in almond and apple [42]. The present work contributes to characterization of the molecular basis for these and other physiological traits of interest to plant breeders.

## Conclusions

The current research provides an overview of the essential processes associated with seed stratification and bud endodormancy, and a description of possible impediments that may result in dormancy. The results show that ER stress and the UPR in seeds and buds elicit BiP chaperones. Thus, both seed and bud endodormancy involve a complex network of signalling. We conclude that endogenous dormancy of seeds and buds is associated with ER stress and the UPR, and the ER stress signal pathway could play a pivotal role in the complex network of endogenous dormancy regulation. The emerging interplay between seed stratification and bud endodormancy status suggests that these two fundamental processes are probably regulated by common signalling pathways. The identification of ER stress and UPR signal pathways provides a new perspective for future research to evaluate their significance in dormancy. The elucidation of general regulatory pathways in both seeds and buds may contribute to an improved knowledge of dormancy mechanisms, and may be valuable for plant breeding programs that will profit from early prediction of chilling requirements for blooming of novel genotypes.

## References

[1] Cooke JE, Eriksson ME, Junttila O (2012) The dynamic nature of bud dormancy in trees: environmental control and molecular mechanisms. Plant Cell Environ 35: 1707–1728. 22670814 10.1111/j.1365-3040.2012.02552.x

[2] Bewley JD (1997) Seed germination and dormancy. Plant Cell 9: 1055–1066. 12237375 10.1105/tpc.9.7.1055 PMC156979

[3] Horvath DP, Anderson JV, Chao WS, Foley ME (2003) Knowing when to grow: signals regulating bud dormancy. Trends Plant Sci 8: 534–540. 14607098 10.1016/j.tplants.2003.09.013

[4] Zhuang WB, Shi T, Gao ZH, Zhang Z, Zhang JY (2013) Differential expression of proteins associated with seasonal bud dormancy at four critical stages in Japanese apricot. Plant Biol 15: 233–242. 22672637 10.1111/j.1438-8677.2012.00589.x

[5] Jimenez S, Reighard GL, Bielenberg DG (2010) Gene expression of DAM5 and DAM6 is suppressed by chilling temperatures and inversely correlated with bud break rate. Plant Mol Biol 73: 157–167. 20143130 10.1007/s11103-010-9608-5

[6] Yamane H, Kashiwa Y, Ooka T, Tao R, Yonemori K (2008) Suppression subtractive hybridization and differential screening reveals endodormancy-associated expression of an SVP/AGL24-type MADS-box gene in lateral vegetative buds of Japanese apricot. J Am Soc Hortic Sci 133: 708–716.

[7] Sreekantan L, Mathiason K, Grimplet J, Schlauch K, Dickerson JA, et al (2010) Differential floral development and gene expression in grapevines during long and short photoperiods suggests a role for floral genes in dormancy transitioning. Plant Mol Biol 73: 191–205. 20151315 10.1007/s11103-010-9611-x

[8] Ruttink T, Arend M, Morreel K, Storme V, Rombauts S, et al (2007) A molecular timetable for apical bud formation and dormancy induction in poplar. Plant Cell 19: 2370–2390. 17693531 10.1105/tpc.107.052811 PMC2002631

[9] Mazzitelli L, Hancock RD, Haupt S, Walker PG, Pont SD, et al (2007) Co-ordinated gene expression during phases of dormancy release in raspberry (Rubus idaeus L.) buds. J Exp Bot 58: 1035–1045. 17244630 10.1093/jxb/erl266

[10] Katz E, Fon M, Lee YJ, Phinney BS, Sadka A, et al (2007) The citrus fruit proteome: insights into citrus fruit metabolism. Planta 226: 989–1005. 17541628 10.1007/s00425-007-0545-8

[11] Chibani K, Ali-Rachedi S, Job C, Job D, Jullien M, et al (2006) Proteomic analysis of seed dormancy in Arabidopsis. Plant Physiol 142: 1493–1510. 17028149 10.1104/pp.106.087452 PMC1676062

[12] Walter P, Ron D (2011) The unfolded protein response: from stress pathway to homeostatic regulation. Science 334: 1081–1086. 22116877 10.1126/science.1209038

[13] Liu Y, Burgos JS, Deng Y, Srivastava R, Howell SH, et al (2012) Degradation of the endoplasmic reticulum by autophagy during endoplasmic reticulum stress in Arabidopsis. Plant Cell 24: 4635–4651. 23175745 10.1105/tpc.112.101535 PMC3531857

[14] Ron D, Walter P (2007) Signal integration in the endoplasmic reticulum unfolded protein response. Nat Rev Mol Cell Biol 8: 519–529. 17565364 10.1038/nrm2199

[15] Martinez IM, Chrispeels MJ (2003) Genomic analysis of the unfolded protein response in Arabidopsis shows its connection to important cellular processes. Plant Cell 15: 561–576. 12566592 10.1105/tpc.007609 PMC141221

[16] Liu JX, Howell SH (2010) Endoplasmic reticulum protein quality control and its relationship to environmental stress responses in plants. Plant Cell 22: 2930–2942. 20876830 10.1105/tpc.110.078154 PMC2965551

[17] Martinez IM (2003) Genomic analysis of the unfolded protein response in Arabidopsis shows its connection to important cellular processes. Plant Cell 15: 561–576. 12566592 10.1105/tpc.007609 PMC141221

[18] Humbert S, Zhong SH, Deng Y, Howell SH, Rothstein SJ (2012) Alteration of the bZIP60/IRE1 pathway affects plant response to ER stress in Arabidopsis thaliana. PLoS One 7: e39023. 22701744 10.1371/journal.pone.0039023 PMC3373542

[19] Iwata Y, Fedoroff NV, Koizumi N (2008) Arabidopsis bZIP60 is a proteolysis-activated transcription factor involved in the endoplasmic reticulum stress response. Plant Cell 20: 3107–3121. 19017746 10.1105/tpc.108.061002 PMC2613661

[20] Liu JX, Srivastava R, Che P, Howell SH (2007) Salt stress responses in Arabidopsis utilize a signal transduction pathway related to endoplasmic reticulum stress signaling. Plant J 51: 897–909. 17662035 10.1111/j.1365-313X.2007.03195.x PMC2156172

[21] Liu JX, Srivastava R, Che P, Howell SH (2007) An endoplasmic reticulum stress response in Arabidopsis is mediated by proteolytic processing and nuclear relocation of a membrane-associated transcription factor, bZIP28. Plant Cell 19: 4111–4119. 18156219 10.1105/tpc.106.050021 PMC2217655

[22] Srivastava R, Deng Y, Shah S, Rao AG, Howell SH (2013) BINDING PROTEIN is a master regulator of the endoplasmic reticulum stress sensor/transducer bZIP28 in Arabidopsis. Plant Cell 25: 1416–1429. 23624714 10.1105/tpc.113.110684 PMC3663277

[23] Fujita M, Mizukado S, Fujita Y, Ichikawa T, Nakazawa M, et al (2007) Identification of stress-tolerance-related transcription-factor genes via mini-scale Full-length cDNA Over-eXpressor (FOX) gene hunting system. Biochem Biophys Res Commun 364: 250–257. 17937930 10.1016/j.bbrc.2007.09.124

[24] Che P, Bussell JD, Zhou W, Estavillo GM, Pogson BJ, et al (2010) Signaling from the endoplasmic reticulum activates brassinosteroid signaling and promotes acclimation to stress in Arabidopsis. Sci Signal 3: ra69. 20876872 10.1126/scisignal.2001140

[25] Deng Y, Humbert S, Liu JX, Srivastava R, Rothstein SJ, et al (2011) Heat induces the splicing by IRE1 of a mRNA encoding a transcription factor involved in the unfolded protein response in Arabidopsis. Proc Natl Acad Sci USA 108: 7247–7252. 21482766 10.1073/pnas.1102117108 PMC3084119

[26] Gao H, Brandizzi F, Benning C, Larkin RM (2008) A membrane-tethered transcription factor defines a branch of the heat stress response in Arabidopsis thaliana. Proc Natl Acad Sci USA 105: 16398–16403. 18849477 10.1073/pnas.0808463105 PMC2571009

[27] Valente MAS, Faria JA, Soares-Ramos JR, Reis PA, Pinheiro GL, et al (2009) The ER luminal binding protein (BiP) mediates an increase in drought tolerance in soybean and delays drought-induced leaf senescence in soybean and tobacco. J Exp Bot 60: 533–546. 19052255 10.1093/jxb/ern296 PMC2651463

[28] Jia XY, Xu CY, Jing RL, Li RZ, Mao XG, et al (2008) Molecular cloning and characterization of wheat calreticulin (CRT) gene involved in drought-stressed responses. J Exp Bot 59: 739–751. 18349049 10.1093/jxb/erm369

[29] Leida C, Conejero A, Arbona V, Gomez-Cadenas A, Llacer G, et al (2012) Chilling-dependent release of seed and bud dormancy in peach associates to common changes in gene expression. PLoS One 7: e35777. 22590512 10.1371/journal.pone.0035777 PMC3349671

[30] Leida C, Terol J, Marti G, Agusti M, Llacer G, et al (2010) Identification of genes associated with bud dormancy release in Prunus persica by suppression subtractive hybridization. Tree Physiol 30: 655–666. 20231169 10.1093/treephys/tpq008

[31] Anderson JV, Chao WS, Horvath DP (2001) Review: A current review on the regulation of dormancy in vegetative buds. Weed Sci 49: 581–589.

[32] Heide O, Prestrud A (2005) Low temperature, but not photoperiod, controls growth cessation and dormancy induction and release in apple and pear. Tree Physiol 25: 109–114. 15519992 10.1093/treephys/25.1.109

[33] Arora R, Rowland LJ, Tanino K (2003) Induction and release of bud dormancy in woody perennials: A science comes of age. HortScience 38: 911–921.

[34] Otero JH, Lizak B, Hendershot LM (2010) Life and death of a BiP substrate. Semin Cell Dev Biol 21: 472–478. 20026282 10.1016/j.semcdb.2009.12.008 PMC2883687

[35] Leborgne-Castel N, Jelitto-Van Dooren EP, Crofts AJ, Denecke J (1999) Overexpression of BiP in tobacco alleviates endoplasmic reticulum stress. Plant Cell 11: 459–470. 10072404 10.1105/tpc.11.3.459 PMC144191

[36] Koiwa H, Li F, McCully MG, Mendoza I, Koizumi N, et al (2003) The STT3a subunit isoform of the Arabidopsis oligosaccharyltransferase controls adaptive responses to salt/osmotic stress. Plant Cell 15: 2273–2284. 12972670 10.1105/tpc.013862 PMC197294

[37] Alvim FC, Carolino SM, Cascardo JC, Nunes CC, Martinez CA, et al (2001) Enhanced accumulation of BiP in transgenic plants confers tolerance to water stress. Plant Physiol 126: 1042–1054. 11457955 10.1104/pp.126.3.1042 PMC116461

[38] Iwata Y, Sakiyama M, Lee MH, Koizumi N (2010) Transcriptomic response of Arabidopsis thaliana to tunicamycin-induced endoplasmic reticulum stress. Plant Biotechnol 27: 161–171.

[39] Liu JX, Howell SH (2010) bZIP28 and NF-Y transcription factors are activated by ER stress and assemble into a transcriptional complex to regulate stress response genes in Arabidopsis. Plant Cell 22: 782–796. 20207753 10.1105/tpc.109.072173 PMC2861475

[40] Reis PA, Rosado GL, Silva LA, Oliveira LC, Oliveira LB, et al (2011) The binding protein BiP attenuates stress-induced cell death in soybean via modulation of the N-rich protein-mediated signaling pathway. Plant Physiol 157: 1853–1865. 22007022 10.1104/pp.111.179697 PMC3327224

[41] Noh SJ, Kwon CS, Oh DH, Moon JS, Chung WI (2003) Expression of an evolutionarily distinct novel BiP gene during the unfolded protein response in Arabidopsis thaliana. Gene 311: 81–91. 12853141 10.1016/s0378-1119(03)00559-6

[42] Mehlenbacher SA, Voordeckers AM (1991) Relationship of flowering time, rate of seed-germination, and time of leaf budbreak and usefulness in selecting for late-flowering apples. J Am Soc Hortic Sci 116: 565–568.

